# Supplementary material for: Chronic High-Fat Diet Induces Early Barrett’s Esophagus in Mice through Lipidome Remodeling
Source: Biomolecules. 2020 May 16;10(5):776. doi: 10.3390/biom10050776 (PMC7277507; doi:10.3390/biom10050776)
Supplement: Supplementary file 1 [file biomolecules-10-00776-s001.zip › Supplemental Info/Table S2_V2.docx]

**Table S2.** Pathology evaluation of inflammation and morphology of gastro-esophageal junction sections following 9 months of dietary intervention.

|  | | **Group** | **Chow** | | **HFD** | |
| --- | --- | --- | --- | --- | --- | --- |
|  |  |  | **Water** | **DCA** | **Water** | **DCA** |
| Pathology | Number of mice | | 11 | 11 | 11 | 11 |
| Inflammation | No | Count | 9 | 9 | 8 | 5 |
|  |  | % of group | 81.8% | 81.8% | 72.7% | 45.5% |
|  | Yes | Count | 2 | 2 | 3 | 6 |
|  |  | % of group | 18.2% | 18.2% | 27.3% | 54.5% |
|  | Mild, grade 1 | Count | 2 | 2 | 2 | 4 |
|  |  | % of group | 18.2% | 18.2% | 18.2% | 36.4% |
|  | Moderate, grade 2 | Count | 0 | 0 | 1 | 1 |
|  |  | % of group | 0.0% | 0.0% | 9.1% | 9.1% |
|  | Severe, grade 3 | Count | 0 | 0 | 0 | 1 |
|  |  | % of group | 0.0% | 0.0% | 0.0% | 9.1% |
| Metaplasia | No | Count | 10 | 10 | 7 | 8 |
|  |  | % of group | 90.9% | 90.9% | 63.6% | 72.7% |
|  | Yes | Count | 1 | 1 | 4 | 3 |
|  |  | % of group | 9.1% | 9.1% | 36.4% | 27.3% |
|  | ≤3mm | Count | 0 | 0 | 2 | 0 |
|  |  | % of group | 0.0% | 0.0% | 18.2% | 0.0% |
|  | >3mm to ≤6mm | Count | 0 | 1 | 1 | 0 |
|  |  | % of group | 0.0% | 9.1% | 9.1% | 0.0% |
|  | >6mm | Count | 1 | 0 | 1 | 3 |
|  |  | % of group | 9.1% | 0.0% | 9.1% | 27.3% |
